# Health Behaviors among Male and Female University Students in Cambodia: A Cross-Sectional Survey

**DOI:** 10.1155/2020/6740236

**Published:** 2020-03-11

**Authors:** Say Sok, Khuondyla Pal, Sovannary Tuot, Rosa Yi, Pheak Chhoun, Siyan Yi

**Affiliations:** ^1^KHANA Center for Population Health Research, Phnom Penh, Cambodia; ^2^Department of Media and Communication, Royal University of Phnom Penh, Phnom Penh, Cambodia; ^3^Faculty of Development Studies, Royal University of Phnom Penh, Phnom Penh, Cambodia; ^4^Center for Global Health Research, Touro University California, Vallejo, CA, USA; ^5^Saw Swee Hock School of Public Health, National University of Singapore and National University Health System, Singapore; ^6^School of Public Health, National Institute of Public Health, Phnom Penh, Cambodia

## Abstract

Students go through a transition when they enter university, which involves major individual and contextual changes in every domain of life that may lead to several behavioral and health problems. This study examined a wide range of health behaviors and practices among 1,359 male and female students recruited from two public universities in Cambodia using a multistage cluster sampling method. Health-related information in different domains were collected using a structured questionnaire. We compared the variables in male and female students. Of the total, 50.8% were male and the mean age was 21.3 (SD = 2.3) years. The majority (79.5%) reported not having any vigorous-intensity activities, 25.9% not having moderate-intensity activities, and 33.5% not having walked continuously for 10 min over the last week. More than one-third (38.3%) reported drinking alcohol, 1.1% smoking tobacco, and 0.4% using an illicit drug in the past 12 months. About one in ten (10.6%) reported having sexual intercourse; of whom, 42.4% reported not using a condom in the last intercourse, and the mean number of sexual partners was 2.1 (SD = 2.4) in the past 12 months. Only 7.1% reported having been diagnosed with a sexually transmitted infection (STI) in the past 12 months; of whom, 60% sought for treatment for the most recent STI. About one-third (33.6%) reported eating fast food at least once over the last week. More than half (55.6%) had one to two servings of fruits or vegetables daily, and 9.9% did not eat any fruits or vegetables over the last week. Gender differences were observed in physical activities, dietary intakes, cigarette smoking, alcohol drinking, and sexual behaviors. Findings from this study indicate that public health and education policies should promote healthy behaviors among university students. The interventions may take advantage of and expand upon the positive health behaviors and consider gender differences.

## 1. Introduction

Globally, chronic diseases such as cancer, stroke, heart disease, and diabetes are the major causes of deaths [1]. Behavioral risk factors including tobacco smoking, alcohol consumption, sedentary behaviors, and obesity are major determinants of adult chronic diseases, morbidity, and mortality [1–4]. In addition to the disease burden attributed to single chronic behavioral risk factors, a growing body of evidence suggests that many behavioral risk factors co-occur among youths, including university students [5–7] and that their combinations yield greater risks for chronic noncommunicable diseases (NCDs) than the sum of their individual independent effects [8, 9].

People living in developing countries are mostly affected by health risks and behaviors associated with poverty such as undernutrition and unsafe sex [10]. With economic growth and the increase in life expectancies, major risks to health have shifted from these risks to other more contemporary risks, e.g., obesity due to sedentary lifestyle, other diet-related factors such as fast food and sugary product consumption, as well as tobacco and alcohol use [10, 11]. Therefore, besides coping with the heavy burden of diseases associated with underdevelopment, these countries need to fight with the growing burden of NCDs as well [10, 11].

Different health risk behaviors have been shown to be associated with several important factors, including economic growth, mobility, and psychological well-being. Studies have shown that economic growth can lead to a rise in obesity or over nutrition, which suggests that people aiming for higher economic mobility may be at risk for obesity [12]. Residential mobility and low self-esteem have been reported to be associated with health risk behaviors, including engagement in early sexual debut and poor eating habits in adolescents [13]. Young populations therefore need to be aware of the myriads of health risks for diseases in order to prevent further incidence [14]. There are also many predictors for mental health problems and suicidal behaviors that include substance abuse, lack of social support, negative family environment, major life stressors, peer pressure and conformity, and demographic factors [15, 16].

Health behaviors formed during early life can have a significant impact on the occurrence of future illnesses and may continue into adulthood and beyond [17, 18]. While the full etiology of these conditions has yet to be understood, behavioral factors during childhood and adolescence such as physical inactivity, tobacco and alcohol use, and unhealthy dietary intake are strongly implicated as risk and protective factors of several physical and psychological well-being of the populations [3, 19, 20]. The rationale for conducting research on university students is that this population is faced with a lot of transitions in their academic and living environments [7], which may lead to either positive or negative health behaviors. They need to adapt to new changes, brought about by the academic transition, and have more freedom over their health and lifestyle. This important transition provides a good opportunity to adopt healthy behaviors or otherwise [21].

Like the trend in the general youths, university students are exposed to several health risk behaviors. Research has shown that university students throughout the world engage in health risk behaviors, and such can have long-term implications for their health and lifestyles [6, 17, 18, 22]. Heavy drinking and other health risk behaviors, for example, tend to increase during this academic transition [23]. Many studies from around the world have identified different health risk behaviors among university students such as smoking, use of alcohol and illicit drugs, risky sexual behaviors, unhealthy eating, poor weight control, and the lack of practice of physical exercises [6, 7, 17, 24–26]. Like anywhere else, students in Cambodia go through the same transition when they enter a university, which involves major individual and contextual changes in every domain of life. However, there is no known study that examines health risk behaviors among Cambodian university students. A few studies on risky sexual behaviors and substance use among other Cambodian youth groups, however, indicate that some health risk behaviors are rather a concern [27–30]. Unmarried young adults, for example, are more prone to engage in unsafe sexual intercourse, and high school adolescents began to engage in social drinking, early sexual debut, and to a lesser extent cigarette smoking [27, 30].

Studies of health behaviors, especially of university students, in low- and middle-income countries are important since the mortality and morbidity from common causes such as coronary heart disease and cancers and adoption of healthy lifestyles differ widely across countries and cultures [6, 7, 10, 31], and not much is known about the situation in Cambodia. The National Institute for Health and Care Excellence, the United Kingdom, stressed that social and economic conditions can prevent people from changing their behaviors to improve their health and can also reinforce behaviors that damage it [32]. Besides the great disparity of social and economic conditions within the country, being one of the fastest growing economies would intensify the impact of social and economic conditions on lifestyle and behaviors of young populations in Cambodia.

University students will occupy important positions in the government and society, including ones that promote public health. It is therefore important to understand their health behaviors and to promote remedial actions [7]. The aim of this study is to assess a wide range of health behaviors among university students in Cambodia. Specifically, the article focuses on six areas of basic health behaviors: physical activities, substance use, sexual behaviors, eating behaviors, sleeping behaviors, and motor-vehicle driving behaviors.

## 2. Materials and Methods

### 2.1. Study Design and Settings

This is a cross-sectional study, and the Strengthening the Reporting of Observational Studies in Epidemiology (STROBE) guideline was followed. Data were collected in June and July 2015 from a sample of undergraduate students randomly selected from the Royal University of Phnom Penh and University of Battambang. These universities were purposively selected to partake in this research. The Royal University of Phnom Penh is the oldest and largest university in Cambodia, and the University of Battambang is one of the most established universities in northwestern Cambodia.

### 2.2. Sample Size

Epi Info (Atlanta, GA, United States) was used to calculate the sample size. There were approximately 168,000 students registered in the higher education institutions in 2013 [33]. Because the prevalence of health risk behaviors among university students in Cambodia was not known, a 50% rate was used for the calculation to prevent any underestimated prevalence. Based on a 95% confidence level (CI) and a 5% margin of error, the minimum required sample size was 767 students. Adjusted for 10% of incomplete response, missing data, and refusal rate, the final minimum required sample size was 850.

### 2.3. Sampling and Data Collection

A multistage cluster sampling method was used to select participants. First, two universities were conveniently selected for administration and logistics purposes. Undergraduate students from first to fourth years from all departments in the two universities were eligible and selected to participate in the study. In each department, a proportional-to-size sampling method was used to select the students to meet the required sample size. Participants were randomly selected from a name list of students in each department, and a personal identification number was assigned to each selected student. On the designated date of data collection, all selected students were approached by our trained data collectors, and questionnaires with an information sheet were delivered to them. Students were asked for written informed consent.

### 2.4. Variables and Measurements

A structured questionnaire was developed in English, translated into Khmer, and back translated into English by the local bilingual research team. The questionnaire was pretested as part of data collection training with 10 male and 10 female students at the Royal University of Phnom Penh, who were later excluded from the main study. The final questionnaire contained 82 items, which took approximately 20 to 30 min to complete.

The design of the core parts of the self-reported questionnaire was informed by the Health Behavior Survey [34], the National College Health Risk Behavior Survey (the United States) [35], and the Global School-based Student Health Survey [36]. There were five sections in the survey. The first section collected information on sociodemographic characteristics of the participants including age (continuous), gender (male, female), marital status (single, married, cohabiting), year of study in the university, living situation (with parent, relative, friend, partner, or alone), perceived family economic status (wealthy, quite well-off, not very well-off, and quite poor), and academic performance (excellent, good, fair, and poor). The second section included questions on vigorous- and moderate-intensity physical activities during the last seven days using the International Physical Activity Questionnaire short version (IPAQ-S7S) [37].

Substance use was covered in the third section. Variables included current use of tobacco (yes, no), experience of daily tobacco use (yes, no), frequency of smoking (not smoking, once or twice per week, weekly), current status of alcohol drinking (nondrinker, occasional drinker, and regular drinker), average number of alcohol drinks during the past two weeks (cans or bottles for beer and glasses for wine, continuous), and the use of illicit drugs (yes, no) during past 12 months.

Sexual behaviors were the focus of the fourth section. Participants were asked questions on whether they had sexual intercourse in the past 12 months (yes, no), age at first sexual intercourse (continuous), number of sexual partners in the past 12 months (continuous), alcohol and drug use during the last sex (yes, no), condom use during the last sex (yes, no), sex in exchange for money or gifts in the past 12 months (yes, no), condom use during the last sex in exchange for money or gifts, diagnosis of sexually transmitted infections (STIs) in the past 12 months (yes, no), treatment seeking behaviors for the most recent STI, and history of unwanted pregnancy in lifetime.

Eating, sleeping, and driving behaviors were covered in Section 2.4. Overall, the variables in this section collected information on the frequency and amount of fast food, soft drinks, soda or sweet tea, high-fat snacks, margarine, butter, meat fat, fruits or vegetables, and lean protein consumed during the past week. We also asked about the average sleeping hours in 24 hours during the past week (continuous).

### 2.5. Data Analyses

Data were cleaned and entered into a Microsoft Excel (version 2010) database and analyzed using SPSS version 22.0 (IBM Corporation, New York, USA). Descriptive statistics were used to compute means and standard deviations for continuous variables as well as frequencies and percentages for categorical variables. Exploratory univariate and bivariate analyses were conducted to measure the distribution of frequencies of variables. Bivariate analyses were conducted using chi-squared test (or Fisher's exact test when a sample size was smaller than five in one cell) for categorical variables and independent Student's *t*-test for continuous variables to compare sociodemographic characteristics, health status, and health risk behaviors by student sex.

### 2.6. Ethical Considerations

The National Ethics Committee for Health Research of the Ministry of Health, Cambodia, approved the study protocol and materials (No. 191NECHR). Participation in this study was voluntary. In the process of obtaining their written informed consent, participants were made clear that they could refuse or discontinue their participation at any time and for any reason. The confidentiality and privacy of the respondents were protected by administering the questionnaires in a private premise and by excluding personal identifiers in the survey.

## 3. Results

Of 1,462 students approached, 1,359 completed the questionnaire (a response rate of 92.9%). The majority (98.6%) of those who declined the participation reported time constraint as the main reason. As shown in Table 1, 50.8% of the respondents were male and the mean age was 21.3 (SD = 2.3) years. The majority (97.9%) of the respondents were single. Of the total, 36.5% was in year one and 70.8% reported fair academic performance. Less than half (43.8%) were living with parents, and 67.3% reported coming from a not very well-off or quite poor family. Compared with females, male students were significantly older. A significantly higher proportion of male students lived with friends or alone and perceived that their families were not well-off. Female students were significantly more likely to live with their parents and perceive that their families were quite well-off.

As shown in Table 2, 79.5% of the respondents had no vigorous-intensity activities; 25.9% had no moderate-intensity activities; and 33.5% did not walk continuously for 10 min in the past seven days. More than half of those who had vigorous-intensity activities (51.1%) and those who had moderate-intensity activities (59.6%) did it in a period of less than 30 min. Of those who had walked continuously for at least 10 min, 73.1% did it in a period of less than 30 min. Compared with males, a significantly higher proportion of female students reported never having vigorous-intensity activities and having ≤30 min of vigorous-intensity activities.

Table 3 shows that 38.3% of the respondents reported being current alcohol drinkers, 1.1% smoking tobacco, and 0.4% using illicit drugs in the past 12 months. Of those who reported using illicit drugs, 50.0% reported using methamphetamines (locally known as yama, yaba, or ice), 25.0% using heroin, and 25.0% using other types of substances as the main drug in the past 12 months. Among those who reported alcohol drinking, average number of days they drank in the past two weeks was 1.3 (SD = 2.0), and average number of alcoholic drinks used in the past two weeks was 1.8 (SD = 2.9). Compared with females, a significantly higher proportion of male students reported smoking tobacco in the past three months and described themselves as occasional or regular drinkers. A significantly higher proportion of male students reported drinking more often and drinking higher amount of alcohol in the past two weeks.

As shown in Table 4, 10.6% of the respondents reported having sexual intercourse in the past 12 months with an average number of sexual partners of 2.1 (SD = 2.4). Among those sexually active respondents, 42.4% reported not using a condom and 6.3% reported using alcohol during their last sexual intercourse. Only 9.2% reported having sex in exchange for money or gifts. Less than one in 10 (7.1%) reported having been diagnosed with an STI in the past 12 months; of whom, 60.0% sought for treatment for the most recent STI. Among students who reported having sexual intercourse in the past 12 months, 12.7% reported having been or made someone pregnant in lifetime. Compared with females, a significantly higher proportion of male students reported having sexual intercourse, having more sexual partners, using a condom in the last sexual intercourse, and having sex in exchange for money or gifts in the last 12 months.

Table 5 presents eating and driving behaviors among the participants. Of the total, 33.6% reported having fast food once or twice per week and 5.3% having it three times or more per week. More than half (57.3%) reported drinking soft or sweet drinks once or twice per week and 26.0% drinking three times or more per week. About two-thirds (63.9%) reported consuming high-fat snacks once or twice per week and 13.5% consuming three times or more per week. Similarly, 51.2% reported consuming fat and its associated products daily, and 7.2% consuming a lot of them. About one in ten (9.9%) reported not consuming any fruits or vegetables and 12.1% not consuming any lean meat in the past week. On average, participants reported sleeping 7.2 hours per day (SD = 1.5), while 33.4% reported having mild sleeping problems, and 50.8% having moderate or severe sleeping problems. Of those who used a car in the past 12 months, 22.3% never or rarely used a seatbelt. Of those who used a motorbike, 6.0% never or rarely used a helmet. Regarding drunk driving, 14.0% reported driving or riding a vehicle after drinking at least once in the past 30 days. Compared with males, female students were significantly more likely to report eating fast food and high-fat snacks. Female students were significantly less likely to consume soft drinks, soda, or sweet tea and more likely to consume fruits and vegetables. Female students were significantly more likely to perceive that they were overweight and less likely to report driving a car or motor vehicle after drinking alcohol in the past 30 days.

## 4. Discussion

This study is among the few investigations into health behaviors of Cambodian university students. There were a comparable number of male and female respondents, and they came from all years of the undergraduate programs in two large public universities. As discussed below, while many major findings are perhaps more or less in line with findings elsewhere or in other studies of Cambodian youths, there are some differences too.

A positive point about health behaviors of university students in this study is that the majority of the respondents were not involved in any of the major health-risk behaviors, including tobacco use, drug use, heavy alcohol consumption, and risky sexual activities; hence, they could veer from negative outcomes from such behaviors. Some of these findings seem to contradict with or different from those of previous studies conducted on university students or youths, more broadly, in Cambodia [30] or elsewhere [38]. Reproductive health issues and casual sex, for instance, were reportedly higher in previous studies in Cambodia that included both in-school and out-of-school youths [27, 39]. The self-reported consumption of alcohol seemed to be relatively lower than that of adolescents in Cambodia and in some other countries in the region as reported in other studies [6, 40, 41]. This may also be applied to the use of tobacco [42, 43].

Such positive results are good for individual development and public health, given the grave consequences from their consumption or involvement on the individuals' health and finance and on the government and society at large [11, 38]. Drug and alcohol, for instance, are known to be negatively associated with individuals' and family well-being and can incur high societal and economic cost to the government [44]. Drug use and trafficking is a barrier to achieving international development, including the sustainable development goals [44]. A systematic review of alcohol consumption and disease burden found that alcohol affects many disease outcomes (both chronic and acute) and injuries, including deaths [45]. Another systematic review indicated that heavy alcohol consumption in the late adolescence continues into adulthood and is associated with alcohol problems [46].

Besides, there were some positive eating behaviors among the students too. That is, they reported low consumption of fast food, soft and sweet drinks, high-fat snacks, and fat and fat-associated products, quite adequate sleep. The findings on healthy dietary behaviors from other studies are inconclusive. Some studies indicated that students elsewhere have “satisfactory” eating habits, while others reported concerning behaviors [18, 47]. Public health policies in Cambodia should take advantages of and sustain these positive behaviors and try to promote such behaviors beyond their university life.

It is plausible that noticeably low proportions of students in this study were involved in major health-risk behaviors, including tobacco and drug use, heavy alcohol consumption, and risky sexual activities. They were also likely to adopt some positive health behaviors such as low consumption of fast food and high-fat products. However, many other negative health behaviors were concerning. These issues include relatively high consumption of soft and sweet drinks; low consumption of fruits, vegetables, and lean protein; sleep disorders; inconsistent use of a seatbelt and helmet; and drunk driving practices. Low fruit and vegetable consumption, high prevalence of fatty food consumption, inadequate sleep, and drunk driving among university students were also observed in studies elsewhere in Europe, Africa, and Asia [7, 17, 25, 26]. However, Cambodian students seemed to consume fruits and vegetable more frequently than their peers in some other Asian countries [7, 25]. Many of these behaviors can have negative implications for individual health, as many of them are more susceptible to the modern health risks [8, 9, 11, 15, 47], which may result in big financial burden and social costs on the individuals, families, and government in the future.

Another noticeable negative instance was the sedentary lifestyles of many participants. The majority of them never had any moderate- or vigorous-intensity activities and never walked continuously for 10 min or more. For those who had moderate- or vigorous-intensity activities, the majority reported having done so for less than 30 min, the level of daily moderate-intensity activity recommended by the World Health Organization for a healthy lifestyle [48]. Other studies also found that university students had inactive lifestyles [7, 47]. However, students in various European countries were reportedly more physically active than Cambodian students [17], but the latter seemed to be more physically active than their counterparts in a few other Asian countries [7]. Apart from the proactive response from the national policy, Cambodian universities may need to promote healthy lifestyles and habits, for example, through extracurricular activities and social engagement.

Differences between the two genders can be observed in some areas including substance use and sexual risk behaviors. Although the prevalence of tobacco smoking was very low in both genders, in line with findings from other parts of the world, smoking is more common in men [42]. However, the difference in smoking was inconclusive in another study on university students in 13 European countries [17]. Similar patterns were observed in alcohol use—the frequency and amount of alcohol consumption was much higher in male students than that of female students [6, 26]. Regarding sexual behaviors, although comparisons were difficult due to the small sample size, male students were more sexually active with higher number of sexual partners and more likely to be involved in transactional sex. However, among sexually active students, female students were more likely to involve in condomless sex. Further studies are needed to address these shortcomings.

Like their peers elsewhere, female students in this study were observed to have better dietary behaviors and nutritional knowledge, perhaps due to the need to watch over one's weight and stronger beliefs in healthy eating [18, 47]. Female students were more likely to report consuming more fruits and vegetables and less soft and sweet drinks, although they were more likely to report consuming more fast and fatty food and had them more frequently. This gender difference in fatty food consumption globally is not conclusive. For example, while a study in South Africa showed that more female students reported higher fatty food consumption and a research in Thailand reported that female students were more conscious to avoid fat and cholesterol [25, 26]. Another survey with Thai students indicated that female students likewise had better fruit and vegetable consumption [25]. Another study, on the other hand, found that Thai male students were more likely to be overweight [47].

Gender differences can also be seen in other variables including perception of body weight, frequency in usage of seatbelts and helmets, and frequency of drunk driving practices. Male students involved more frequently in doing vigorous-intensity activities and spent longer time to do these activities. Various cross-country, large-scale studies, and country-specific research also consistently showed that male students were more physically active [7, 17, 26, 47]. Given the gender differences in many outcome variables (including negative health risk behaviors), a gender lens may be considered in any potential interventions and policy considerations.

Overall, the negative health behaviors and lifestyles could present multiple health risks, especially the threat from chronic diseases and/or severe health conditions, to the participants in the future [2, 3, 49], depending on the intensity of their current health behaviors, risks, and lifestyles and the changes in behaviors and lifestyles in the future. The health risks associated with economic underdevelopment, that is, according to the family status of a majority of the respondents, e.g., 67.3% of them reported as not very well-off or quite poor, may have a negative repercussion on or implications for their diet and quality of the food consumed, among others. These conditions may expose them to other health risks in the future [6, 11]. Moreover, given the multiple unhealthy and risky health behaviors and lifestyles reported by the students, chances are very high that many of the health risk factors could co-occur among many of them. It is commonly understood that negative health behaviors, attitudes, and lifestyles formed during childhood and adolescence have a profound impact on future health behaviors and lifestyles of the individuals. They will have significant negative impact on the individual health as well as familial economy and will be a big burden for the public policy to address in the future [3, 17, 19, 35].

This study has some limitations. Firstly, the cross-sectional design could capture only a snapshot view of the study population and could not document changes in the variables over time. Given that conduct of student or youth health surveys are a rare phenomenon in Cambodia, findings from this study can provide some pioneering thoughts on the issue. Nevertheless, it must be underlined that many respondents reportedly adopted positive health behaviors and lifestyles, which may co-occur [18], although whether they co-occur was not examined in this study and warrant further analyses and research. Many of the health issues need to be examined into more details—examples include quality and adequacy of the food consumed, quality of sleep, and rigorousness of the physical activities. Other health and associated issues that may be addressed in the future research include mental health, family environment, and peer pressure. Secondly, the self-reported measures may have led to social desirability bias, particularly for sensitive information such as sexual behaviors and substance abuse. Future studies may want to triangulate the data through, for example, adoption of a mixed-method study. Third, the study may benefit from multiple regression analyses and multifactorial association tests, which may shed better light on the results that would provide more nuanced and comprehensive findings. However, given that this paper attempts only to present broad basic description of the students' health behaviors, this is beyond the scope of our study. Finally, this study included a sample of students selected from only two major public universities located in the capital city and a province; therefore, findings from this study may not be generalized to a wider university student population in the country. However, because this is one of the few studies on Cambodian students' health, it can provide policy implications for national health and education policy makers as well as the university community.

## 5. Conclusions

Despite the abovementioned limitations, our study provides preliminary insights into health-related behaviors among university students in Cambodia. We found that the prevalence of some health risks (such as risky sexual behaviors, tobacco use, illicit drug use, and to a lesser extent, alcohol consumption) among university students in this study was plausibly low. However, the rates of many other negative health behaviors (sedentary lifestyle, high consumption of unhealthy and low consumption of healthy foods, and emerging habit of alcohol consumption) were concerning. These health risks may predict later poor health including increased risks of noncommunicable chronic diseases such as diabetes, hypertension, and other cardiovascular disease among this potential population. Further studies are needed to explore factors associated with specific health risk behaviors and effective intervention programs for promoting university student health in Cambodia. Effort may be needed to conduct such surveys on a more regular basis and on a larger scale to better inform public health programs and policies.

## Figures and Tables

**Table 1:** Sociodemographic characteristics of male and female university students.

Variables	Frequency			
	Total *n* (%)	Males *n* (%)	Females *n* (%)	*p* value^*∗*^
Age (in years, mean ± SD)	21.3 ± 2.3	21.7 ± 2.5	21 ± 2.2	<0.001

Marital status				0.88
Single	1330 (97.9)	674 (97.7)	656 (98.1)	
Married/cohabiting	29 (2.1)	16 (2.3)	13 (1.9)	

Year of study in the university				0.15
Year 1	496 (36.5)	245 (35.5)	251 (37.5)	
Year 2	281 (20.7)	130 (18.8)	151 (22.6)	
Year 3	251 (18.5)	137 (19.86)	114 (17.0)	
Year 4	331 (24.3)	178 (25.8)	153 (22.9)	

Living arrangement				<0.001
Parent	595 (43.8)	271 (39.3)	324 (48.4)	
Relative	335 (24.6)	173 (25.1)	162 (24.2)	
Friends	346 (25.5)	188 (27.2)	158 (23.6)	
Couple	21 (1.5)	14 (2.0)	7 (1.0)	
Alone	18 (2.7)	44 (6.4)	18 (2.7)	

Perceived family economic status				<0.001
Quite well-off/wealthy	444 (32.7)	195 (28.4)	249 (37.2)	
Not very well-off	846 (62.3)	449 (65.2)	397 (59.3)	
Quite poor	68 (5.0)	45 (6.5)	23 (3.4)	

Perceived academic performance				0.92
Excellent	57 (4.2)	29 (4.2)	28 (4.2)	
Good	303 (22.3)	157 (22.8)	146 (21.8)	
Faire	962 (70.8)	487 (70.6)	475 (71.0)	
Poor	37 (2.7)	17 (2.5)	20 (3.0)	

Abbreviations: *n*, number; SD, standard deviation. ^*∗*^Chi-square test (or fisher's exact test when a sample size was smaller than five in one cell) was used for categorical variables and independent student's *t* test for continuous variables.

**Table 2:** Physical activities among male and university students in the study.

Variables	Frequency			
	Total *n* (%)	Males *n* (%)	Females *n* (%)	*p* value
Work involved vigorous-intensity activities for ≥10 min continuously				0.001
Never	973 (71.7)	444 (64.4)	529 (79.5)	
Had 1–3 days/week	261 (19.3)	159 (23.1)	102 (15.3)	
Had 4–7 days/week	120 (8.9)	86 (12.5)	34 (5.1)	

Time usually spent doing vigorous-intensity activities on one of those days				0.001
≤30 min	161 (51.1)	89 (42.8)	72 (67.3)	
31–60 min	65 (20.6)	53 (25.5)	12 (11.2)	
>60 min	89 (28.3)	66 (31.7)	23 (21.5)	

Work involved moderate-intensity activities for ≥10 min continuously				0.61
Never	352 (25.9)	185 (26.9)	167 (25.0)	
Had 1–3 days/week	564 (41.6)	278 (40.4)	286 (42.8)	
Had 4–7 days/week	441 (32.5)	226 (32.8)	215 (32.2)	

Time spent doing moderate-intensity activities on one of those days				0.94
≤30 min	514 (59.6)	264 (60.0)	250 (59.1)	
31–60 min	204 (23.6)	104 (23.6)	100 (23.6)	
>60 min	145 (16.8)	72 (16.4)	73 (17.3)	

Days walking for ≥10 min continuously to get to and from places				0.08
Never	470 (34.7)	247 (35.9)	223 (33.5)	
Had 1–3 days/week	338 (24.9)	154 (22.4)	184 (27.6)	
Had 4–7 days/week	547 (40.4)	288 (41.8)	259 (38.9)	

Time usually spent walking on one of those days				0.19
≤30 min	545 (73.1)	261 (70.2)	284 (75.9)	
31–60 min	111 (14.9)	60 (16.1)	51 (13.6)	
>60 min	90 (12.1)	51 (13.7)	39 (10.4)	

^*∗*^Chi-squared test (or fisher's exact test when a sample size was smaller than five in one cell) was used.

**Table 3:** Substance use among male and female university students in the study.

Variables	Frequency			
	Total *n* (%)	Males *n* (%)	Females *n* (%)	*p* value
Current tobacco smokers	15 (1.1)	9 (1.3)	6 (0.9)	0.47
Experience of daily smoking for at least 30 days	8 (0.6)	6 (0.9)	2 (0.3)	0.16
Smoking in the past 3 months	16 (1.2)	14 (2.0)	2 (0.2)	0.002
Would you describe yourself as:				<0.001
Nondrinker	838 (61.7)	274 (39.7)	564 (84.3)	
Occasional drinker	367 (27.0)	292 (42.3)	75 (11.2)	
Regular drinker	154 (11.3)	124 (17.9)	30 (4.5)	
Average number of days drinking alcohol in the past 2 weeks (mean ± SD)	1.2 ± 2.0	1.4 ± 2.2	0.7 ± 2.0	0.001
Average number of alcohol drinks^†^ in the past 2 weeks (mean ± SD)	1.8 ± 3.0	2.1 ± 3.1	1.1 ± 2.2	0.002
Used illicit drugs in the past 12 months	4 (0.3)	3 (0.4)	1 (0.1)	0.33

Abbreviations: *n*, number; SD, standard deviation. ^*∗*^Chi-squared test (or fisher's exact test when a sample size was smaller than five in one cell) was used for categorical variables and independent student's *t* test for continuous variables. ^†^Can was a unit used for beer and glass for wine.

**Table 4:** Sexual behaviors among male and female university students in the study.

Variables	Frequency			
	Total *n* (%)	Males *n* (%)	Females *n* (%)	*p* value
Had sexual intercourse in the past 12 months	144 (10.6)	119 (17.3)	25 (3.7)	<0.001
Alcohol use during last sex	9 (6.3)	8 (6.8)	1 (4.0)	0.60
Condom use during last sex	83 (57.6)	75 (63.0)	8 (32.0)	0.004
Sex in exchange for money/gifts	13 (9.2)	13 (11.2)	0 (0)	0.08
Condom use during last sex in exchange for money or gifts	8 (61.5)	8 (61.5)	N/A	N/A
Diagnosed with a STI in the past 12 months	10 (7.1)	9 (7.8)	1 (4.0)	0.51
Sought for treatment for the most recent STI	6 (60.0)	5 (55.6)	1 (100)	0.39
Ever made someone pregnant/been pregnant	18 (12.7)	14 (12.0)	4 (16.0)	0.58
Age at first sexual intercourse (in years, mean ± SD)	20.6 ± 3.2	20.7 ± 2.9	20.2 ± 4.4	0.47
Number of partner in the past 12 months (mean ± SD)	2.1 ± 2.4	2.3 ± 2.6	1.0 ± 0.2	0.02
Age first made someone pregnant/became pregnant (in years, mean ± SD)	23.1 ± 3.2	23.3 ± 3.4	22.3 ± 2.3	0.65

Abbreviations: *n*, number; SD, standard deviation; STI, sexually transmitted infection. ^*∗*^Chi-squared test (or fisher's exact test when a sample size was smaller than five in one cell) was used for categorical variables and independent student's *t* test for continuous variables.

**Table 5:** Eating, sleeping, and driving behaviors among male and female university students.

Variables	Frequency			
	Total *n* (%)	Male *n* (%)	Female *n* (%)	*p* value
Times do eating fast food per week				<0.001
0 times	881 (61.1)	457 (66.2)	374 (55.9)	
1-2 times	456 (33.6)	201 (29.1)	255 (38.1)	
3 or more times	72 (5.3)	32 (4.6)	40 (6.0)	

Number of soft drinks, glasses of soda or sweet tea consumed per day				0.01
None	226 (16.6)	100 (14.5)	126 (18.8)	
1-2	779 (57.3)	389 (56.4)	390 (58.3)	
3 or more	354 (26.0)	201 (29.1)	153 (22.9)	

Times consuming high-fat snacks per week				0.001
0 times	307 (22.6)	174 (25.2)	133 (19.9)	
1-2 times	869 (63.9)	445 (64.5)	424 (63.4)	
3 or more times	183 (13.5)	71 (10.3)	112 (16.7)	

Amount of margarine, butter, meat fat consumed				0.67
None or very little	565 (41.6)	285 (41.3)	280 (41.9)	
Some	696 (51.2)	351 (50.9)	345 (51.6)	
A lot	98 (7.2)	54 (7.8)	44 (6.6)	

Number of servings of fruits or vegetables consumed per day				0.006
0 servings	134 (9.9)	84 (12.2)	50 (7.5)	
1-2 servings	755 (55.6)	385 (55.8)	370 (55.3)	
3 or more servings	470 (34.6)	221 (32.0)	249 (37.2)	

Times consuming lean protein per week				0.81
0 times	164 (12.1)	83 (12.0)	81 (12.1)	
1-2 times	874 (64.3)	439 (63.6)	435 (65.0)	
3 or more times	321 (21.6)	168 (24.3)	153 (22.9)	

Self-perception on body weight				<0.001
Overweight	408 (30.0)	157 (22.8)	251 (37.5)	
About right	496 (36.5)	251 (36.4)	245 (36.6)	
Underweight	455 (33.5)	282 (40.8)	173 (25.8)	

Problem with sleeping in the last 30 days				0.98
None	215 (15.8)	110 (15.9)	105 (15.7)	
Mild	454 (33.4)	231 (33.5)	223 (33.3)	
Moderate	587 (43.2)	295 (42.8)	292 (43.6)	
Severe	103 (7.6)	54 (7.8)	49 (7.3)	

Used seatbelt when driving a car or other motor vehicle in the past 30 days				0.05
I did not drive	756 (55.6)	397 (57.5)	359 (53.7)	
Never	211 (15.5)	88 (12.8)	123 (18.4)	
Rarely	93 (6.8)	44 (6.4)	49 (7.3)	
Sometimes	108 (7.9)	60 (8.7)	48 (7.2)	
Most of the time	68 (5.0)	41 (5.9)	27 (4.0)	
Always	123 (9.1)	60 (8.7)	63 (9.4)	

Used a helmet when riding a bicycle in the past 30 days				0.06
I did not ride	156 (11.5)	64 (9.3)	92 (13.8)	
Never	49 (3.6)	22 (3.2)	27 (4.0)	
Rarely	33 (2.4)	19 (2.8)	14 (2.1)	
Sometimes	51 (3.8)	29 (4.2)	22 (3.3)	
Most of the time	319 (23.5)	179 (25.9)	140 (25.9)	
Always	751 (55.3)	377 (54.6)	374 (55.9)	

Drove a car or other motor vehicle after drinking alcohol in the past 30 days				<0.001
I did not drive	371 (27.3)	151 (21.9)	220 (32.9)	
0 times	797 (58.6)	370 (53.6)	427 (63.8)	
1 time	120 (8.8)	105 (15.2)	15 (2.2)	
2-3 times	52 (3.8)	48 (7.0)	4 (0.6)	
≥4 times	19 (1.4)	16 (2.3)	3 (0.4)	

Average sleeping hours/24 h during the past week (±SD)	7.2 ± 1.5	7.2 ± 1.5	7.3 ± 1.5	0.15

Abbreviations: *n*, number; SD, standard deviation. ^*∗*^Chi-squared test (or fisher's exact test when a sample size was smaller than five in one cell) was used for categorical variables and independent student's *t* test for continuous variables.

## Data Availability

Data underlying the findings of this study are available upon request from the Principal Investigator (Dr. Siyan Yi) at siyan@doctor.com.
